# Human Detection Using Random Color Similarity Feature and Random Ferns Classifier

**DOI:** 10.1371/journal.pone.0162830

**Published:** 2016-09-09

**Authors:** Miaohui Zhang, Ming Xin

**Affiliations:** 1 Institute of Image Processing and Pattern Recognition, Henan University, Kaifeng, China; 2 Beijing Key Laboratory of Digital Media, School of Computer Science and Engineering, Beihang University, Beijing, China; Tianjin University, CHINA

## Abstract

We explore a novel approach for human detection based on random color similarity feature (RCS) and random ferns classifier which is also known as semi-naive Bayesian classifier. In contrast to other existing features employed by human detection, color-based features are rarely used in vision-based human detection because of large intra-class variations. In this paper, we propose a novel color-based feature, RCS feature, which is yielded by simple color similarity computation between image cells randomly picked in still images, and can effectively characterize human appearances. In addition, a histogram of oriented gradient based local binary feature (HOG-LBF) is also introduced to enrich the human descriptor set. Furthermore, random ferns classifier is used in the proposed approach because of its faster speed in training and testing than traditional classifiers such as Support Vector Machine (SVM) classifier, without a loss in performance. Finally, the proposed method is conducted in public datasets and achieves competitive detection results.

## Introduction

Pedestrian detection is an essential and significant task in the field of computer vision. It provides the fundamental information for many vision-based applications, such as intelligent transportation systems, autonomous robotics, video surveillance systems, etc. However, due to high intra-class visual variations of different heights, weights, clothes, accessories, and even postures, and other challenges including partial occlusions, illumination variations, and viewpoint change, vision based pedestrian detection is a challenging task in the field of computer vision.

As described in detail previously [1], Papageorgiou and Poggio [2] employed Haar features in combination with a polynomial SVM to detect humans in early human detection work. The most representative work can be found in [3]. They trained SVM on histograms of oriented gradients (HOG) features and achieved good performance. Applying a combination of edgelets, HOG descriptors, and covariance descriptors, Wu and Nevatia [4] described a cascade based approach where each weak classifier corresponded to a sub-region within the detection window from which different types of features were extracted. Employing part-based detectors, Mikolajczyk et al. [5] divided the human body into several parts and applied a cascade of detectors for each part. Recently, sparse representation based pedestrian detection and integral histograms with random projection for pedestrian detection were introduced by Yao et al. [6] and Liu et al. [7], respectively.

In recent years, some novel human detectors have been explored, such as Riemannian manifold based human detector [8], deep network based human detector [9], human vision driven features based pedestrian detection [10] etc. Tuzel et al. [5] presented a new algorithm to detect humans in still images by utilizing an ensemble of covariance descriptors, which were projected into a Riemannian manifold. Deep Network based pedestrian detection was explored by Luo et al. [9], which proposed a Switchable Deep Network (SDN) for pedestrian detection; the SDN can automatically learn hierarchical features, salience maps, and mixture representations of different body parts. Motivated by the center-surround mechanism in the human visual attention system, Zhang et al. [10] proposed to utilize average contrast maps to detect pedestrians.

With respect to human detection, standard classification techniques like SVM [2,11] and Adaboost [12–14] have been used. Furthermore, Cheng et al. [15] proposed a cascade classifier combining SVM and AdaBoost. The proposed method can automatically select the SVM or AdaBoost classifier to construct a cascade classifier according to the training samples. Compared to the traditional classifiers, the random ferns classifier has recently been used for image classification [16] and object detection [17] because of its faster speed in training and testing than traditional classifiers such as SVM. In [16], Ozuysal et al. used the random ferns classifier for fast keypoint recognition. They treated the set of all possible appearances of the image patch surrounding a keypoint as a class. The training set for each class is formed by generating a large number of sample images with randomly picked affine deformations. This in turn is accomplished by sampling the deformation parameters from a uniform distribution, which were utilized to train the random ferns classifier. Villamizar et al. [17] presented an efficient rotation invariant object detection approach by employing a boosted combination of random ferns over local HOG.

In addition, random ferns classifier has several special advantages. For example, random ferns classifier can enable different cues (such as color feature and shape feature) to be “effortlessly combined”. When the random ferns classifier is trained by feeding with different features, the effective features, which are discriminative for distinguishing humans from non-humans, will frequently take the same paths down the random-ferns and result in peaks in the posterior distributions; on the other hand, the useless feature will naturally be evenly distributed over the leaf nodes and result in flat posterior distributions, which will not influence the detection result. Therefore, we employ random ferns classifier to determine whether the candidate image patch is human or not in our proposed approach.

Though rarely used in human detection in previous literatures, color feature is recently employed to detect humans in still images. Schwartz et al. [18] described a human detection method that adopted color feature, texture feature and widely used edge feature. In [19], color normalization was used to enhance color edges in the color-based HOG (CHOG) descriptor complementary to the intensity edges in the HOG descriptor. The CHOG features emphasized the edges of the human while attenuating shading on the clothing and clutter edges in the background by color normalization. By computing the sub-region self-similarity on color channels, Walk et al. [20] explored color-based self-similarity features, which consistently improved detection performances by combining with HOG features.

In this paper, we introduce a novel color-based feature, RCS feature, which can be extracted by color histogram similarity computation between randomly selected image patches. In contrast to the surrounding environment, the jacket and trouser colors of single pedestrian have strong self-similarity, respectively. Thus, RCS feature can effectively capture pairwise statistics of spatially localized color distributions, is independent of the actual color of a specific example which may vary from person to person depending on their clothing, and can effectively represent the essential attribute of pedestrians.

HOG-based features have demonstrated remarkable results for human detection showing robustness to illumination and object appearance changes. For example, Laptev [21] employed the HOG descriptors in a boosting structure. Dalal et al. [22] further extended the approach in [3] by combining the HOG descriptors with oriented histograms of optical flow to handle space-time information for moving human detection. According to the idea of multi-resolution feature descriptors, Zhao et al. [23] proposed a new robust edge feature referred to as Enhanced HOG (eHOG). Because the combination of multiple and complementary feature types can help improve performance, HOG-based feature is also employed to complement the RCS feature and captures a different part of the image statistics. A composite descriptor is formed by concatenating RCS feature and HOG-LBF feature (RCS-HOG-LBF) in our proposed approach.

The remainder of this work is organized as follows. Sec. 2 reviews random ferns classifier. Sec. 3 introduces HOG-based local binary feature. The RCS feature is presented in Sec. 4. Experimental results are given in Sec. 5, followed by conclusions in Sec. 6.

## Materials and Methods

### Ethics statement

The paper studies how to detect human in static images only by using computer vision technology. All participants agreed to publish these images and provided their written consent to participate in this study using a paper-based consent form.

### Random Ferns Classifier

Random ferns classifier is a variation on the random forest classifier first introduced in [24] and developed further in [25–26]. They have been applied to object recognition in [27], image classification in [28] and fast keypoints recognition in [16]. The advantage of random ferns classifier is that it enables different cues such as appearance and shape to be effortlessly combined as introduced above.

A common method for human detection is to use a sliding windows approach to search for humans in all possible positions and scales. In this way, the detection problem is transformed into a classification problem. Therefore, given a candidate patch in an image, our task is to assign it to the most likely class. Let *c*_*i*_, *i* = 0,1, be the set of classes such as human or non-human, and let *f*_*j*_, *j* = 1, 2, ⋯*N*, be the set of binary features that will be calculated over the patch that we are trying to classify. Formally, we are looking for,

$${\hat{c}}_{i}=\underset{c_{i}}{\text{arg max}}\;P\left(C=c_{i}|f_{1},f_{2},\cdots ,f_{N}\right)$$ (1)

where *C* refers to the class, our goal is to model the posterior human class probability given a set of *N* features. This can be expressed by the Bayes rule as,

$$P\left(C=c_{i}|f_{1},f_{2},\cdots ,f_{N}\right)=\frac{P\left(f_{1},f_{2},\cdots ,f_{N}|C=c_{i}\right)P\left(C=c_{i}\right)}{P\left(f_{1},f_{2},\cdots ,f_{N}\right)}$$ (2)

Similarly, an equivalent expression may be written for the non-human class. By removing the priors *P*(*f*_*1*_, *f*_*2*_, ⋯, *f*_*N*_), common for all the classes, assuming uniform prior probabilities *P*(*C*), the problem reduces to finding,

$${\hat{c}}_{i}=\underset{c_{i}}{\text{arg max}}\;P\left(f_{1},f_{2},\cdots ,f_{N}|C=c_{i}\right)$$ (3)

But learning the joint likelihood distributions over all features is most likely intractable. Naive Bayes makes the simplifying assumption that features are conditionally independent given the class label,

$$P\left(f_{1},f_{2},\cdots ,f_{N}|C=c_{i}\right)=\overset{N}{\underset{j=1}{\Pi }}P\left(f_{j}|C=c_{i}\right)$$ (4)

However, this independence assumption is usually false, tending to grossly underestimate the true posterior probabilities. To make the problem tractable while accounting for these dependencies, a good compromise is to partition our features into *M* groups of size *S = N/M*. These groups are what we define as ferns, and we compute the joint probability for features in each fern. The conditional probability is as follows,

$$P\left(f_{1},f_{2},\cdots ,f_{N}|C=c_{i}\right)=\overset{M}{\underset{k=1}{\Pi }}P\left(F_{k}|C=c_{i}\right)$$ (5)

where *F*_*k*_ = {*f*_*σ*(*k*, 1)_, *f*_*σ*(*k*, 2)_, ⋯, *f*_*σ*(*k*, *S*)_}, *k* = 1, 2, ⋯ *M*, represents the k’th fern and *σ*(*k*, *j*)is a random permutation function. Hence, we follow a semi-naive Bayesian approach by modeling only some of the dependencies between features.

Furthermore, the class-conditional probabilities *P*(*F*_*m*_|*C* = *c*_*i*_) are estimated for each fern *F*_*m*_ and class *c*_*i*_ in the training phase. For each fern *F*_*m*_, these terms are written as,

$$p_{k,c_{i}}=P\left(F_{m}=k|C=c_{i}\right)=\frac{N_{k,c_{i}}}{N_{c_{i}}}$$ (6)

where $N_{k,c_{i}}$ is the number of training samples of class *c*_*i*_ that evaluate to fern value *k*, *k =* 1, 2, ⋯, 2^*S*^ and $N_{c_{i}}$ is the total number of samples for class *c*_*i*_. However, when the number of samples is not infinitely large, both $N_{k,c_{i}}$ and $p_{k,c_{i}}$ will be zero. To overcome this problem, $p_{k,c_{i}}$ is to be taken as,

$$p_{k,c_{i}}=\frac{N_{k,c_{i}}+N_{r}}{N_{c_{i}}}$$ (7)

where *N*_*r*_ represents a regularization term, which behaves as a uniform Dirichlet prior over feature values. In the following experiment, the parameter *N*_*r*_ is set to 1.

### HOG-based Binary Feature

A Local Binary Feature (LBF) maps an image sample *X* to a boolean space in the form, *f*: *x* → {0, 1}, *x* ∈ *X*, by simple comparison between a pair of image values (e.g pixel intensities). Traditionally, LBF is computed in the image intensity domain yielding successful applications such as the tracking application in [26], image texture descriptions in [29], and fast keypoints recognition in [16]. Furthermore, M.Villamizar et al. [17] extended the same idea and proposed to compute LBF in the HOG domain instead.

**Fig 1:**
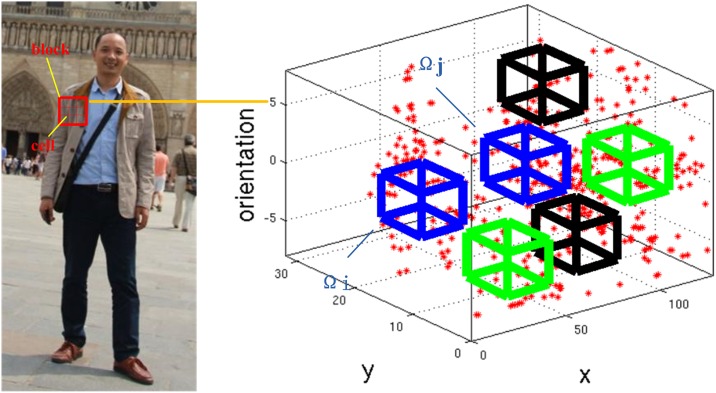
HOG-based Local Binary Feature. The features are computed from binary comparisons between different bins of the HOG.

In our proposed approach, a pre-processing step [19] is firstly performed on both training samples and test images, which can enhance the human/background edges by suppressing textural and shading variations. As a consequence, pedestrian contours are more conspicuous, while reducing clutter from variation in texture and shading. After the pretreatment step, HOG-based local binary feature is defined as a signed comparison between two HOG cells,

$$f(x)=\left\{ \begin{array}{l} 1\;x_{\Omega i}>x_{\Omega j}\\ 0\;x_{\Omega i}\leq x_{\Omega j} \end{array}\right.,\;\Omega \in \text{IR3}$$ (8)

where Ω*i* and Ω*j* are the feature component locations defined by spatial and orientation bin coordinates (*u*, *v*, *θ*). Fig 1 displays one LBF instance for a local HOG.

### Random Color Similarity Feature

**Fig 2:**
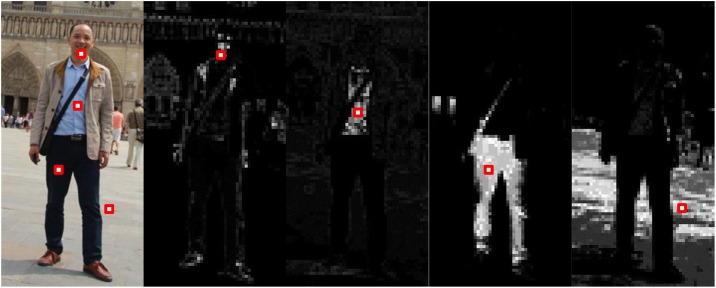
Color similarity computed at marked cell positions. Cells with higher similarity are brighter.

A considerable number of literature has reported improvements by combining multiple types of low-level features. However, color information is popular in object tracking, image classification and recognition but rarely used in human detection because of large intra-class variations. Since humans in standing positions have bilateral symmetry, the color distributions on the left and right shoulder usually exhibit high similarity. In addition, the jacket and trouser colors of a single pedestrian also have strong self-similarity respectively. For example, as illustrated in Fig 2, different parts of the pedestrian body have strong self-similarity. A similar work was done in [20], in which the color feature is generated by exhaustively picking all the cell pairs, but it’s computationally formidable, and the components of feature vector are not independent of each other, so it cannot satisfy the independence requirement for random ferns classifier.

**Fig 3:**
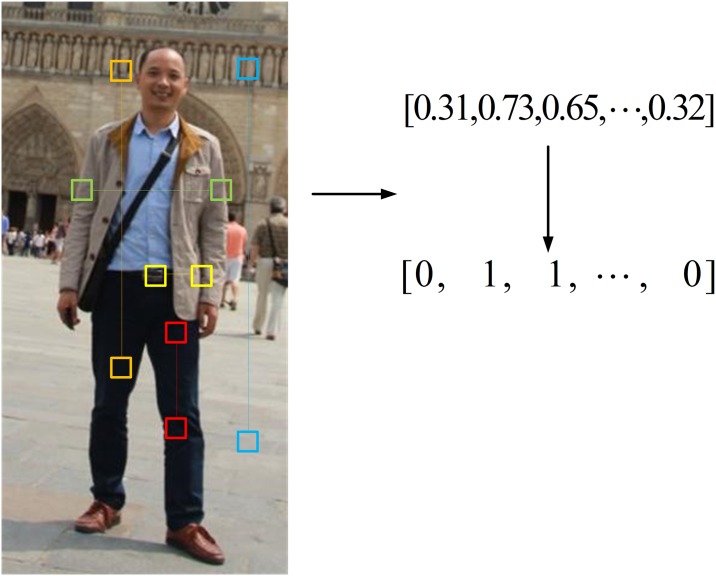
Color based binary feature calculation of RCS feature.

In this section, we introduce a novel color-based feature, RCS feature, which is yielded by simple color similarity computation between image cells randomly picked in pedestrian images. Fig 3 illustrates the computation process. The detailed process is as follows. First, *N* pairwise cells are randomly picked in a pedestrian image, and the cell size is set to 7x7 pixels. The distance between any pairwise cells will be at least 4 pixels. Second, for each cell, a 16×16×16-bin histogram is extracted in RGB color space. The normalized color histogram of each cell is denoted by

$$H={\left\{H^{\left(u\right)}\right\}}^{u=1,2,\cdots m}$$ (9)

where m is the number of bins. Third, for each set of pairwise cells, the similar measure is denoted as follow.

$$\rho_{i}\left[H_{0},H_{1}\right]=1-\sqrt{\sum_{u=1}^{m}{\left(H_{0}^{\left(u\right)}-H_{1}^{\left(u\right)}\right)}^{2}}$$ (10)

where *ρ*_*i*_ is the similarity coefficient of the i’th cell pair, *i =* 1, 2, ⋯, *N*,$H_{0}^{\left(u\right)}$ and $H_{1}^{\left(u\right)}$ represent the normalized color histogram of pairwise cells, respectively. Hence, an *N* -dimensional vector is obtained by computing the color similarity between *N* pairwise cells. Fourth, with respect to the random ferns classifier, both the training features and the test features must be binary features. Therefore, a binary feature mapping criterion is as follows,

$$\rho_{i}=\left\{ \begin{array}{l} 1\;\rho_{i}\geq 0.5\\ 0\;\rho_{i}<0.5 \end{array}\right.\;,i=1,2,\cdots ,N$$ (11)

Now, an *N* -dimensional binary feature vector is yielded by extracting RCS features.

Finally, both the color-based RCS binary features and the HOG-based local binary features are concatenated into a single multi-cue fused feature vector, which is fed to the random ferns classifier for training or classification.

Since both color information and gradient information are combined by our proposed method, the appearance of the human body is better represented. By using RCS-HOG-LBF and the random ferns classifier, the proposed human detection algorithm achieves desired performance. Detailed experimental results are shown in section 5.

## Experimental Results

To evaluate the proposed human detection approach, we use a challenging data set, the INRIA person data set. The data set contains people standing in different positions with different orientations and poses. The training set contains 2416 human images and 1218 non-human images. The testing set contains 1132 human images and 453 non-human images. In the next verification experiment, the detector is initially trained using (i) cropped and resized images of humans as positive samples and (ii) randomly cropped and resized image regions not containing humans as negative samples. The sample size is set as 110x40 pixels, the number of positive training samples is 2000, and the number of negative training samples is 500.

For computing gradients, Prewitt masks have been selected and their signs omitted to have unsigned gradients (0°, 180°). In order to reduce the computation cost, an integral histogram strategy is employed. For HOG-based local binary feature computation, the cell size and local HOG size (block size) are set to 3x3 pixels and 3x3 cells, respectively. The number of gradient orientation bins is set to 4. In our approach, the blocks are randomly picked from the sample image. With respect to each block, a 3x3x4-D histogram of oriented gradient feature is yielded according to the configuration properties proposed above. Therefore, HOG-based local binary features are derived by comparing between a few pairwise HOG cells randomly selected from the 36-D HOG features vector. In the next experiment, a total of 100 blocks will be randomly picked from one sample image. For each block, 10-dimensional binary feature vector is yielded by comparing 10 pairwise bins value randomly selected from the 36-D HOG features vector. Therefore, for each sample image, 100x10 HOG based binary features are yielded according to the binarization strategy illustrated as Fig 1.

For computing RCS feature, the basic parameters are set as follows. 300 pairwise cells are randomly picked from the sample image, and the cell size is set as 7x7 pixels. For each cell, the color histogram is computed according to the configuration in which RGB color space is quantized into 16×16×16 bins. Simultaneously, the "integral image" technique is also used to improve the computation efficiency. Then, the similar coefficient is derived by computing the similarities between pairwise cells. Finally, a 300-dimensional color based binary feature vector is yielded by Eq 11.

**Fig 4:**
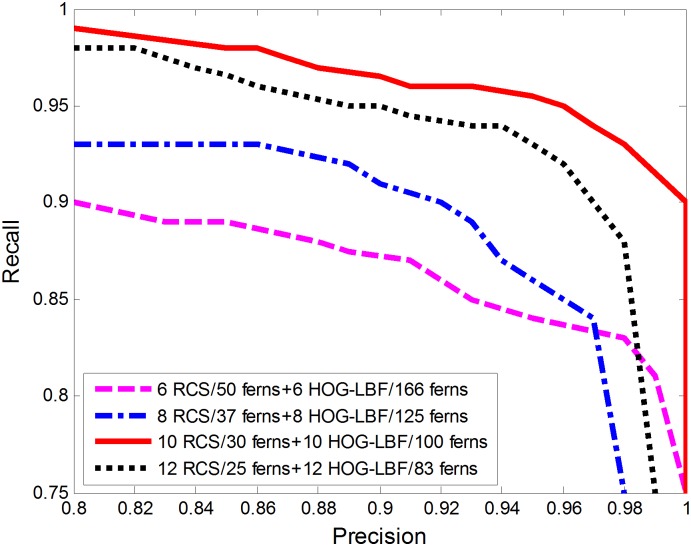
Features and ferns co-occurrence evaluation.

Before classification, we first determine the size of each fern by observing the classifying results when trying various fern sizes. The number of features per fern has been evaluated to measure the importance of feature co-occurrence. Fig 4 shows the performances as the size of each fern increases from 6 to 12. With respect to each sample image, 300-dimensional RCS feature and 1000-dimensional HOG-LBF feature are yielded by the proposed approaches. Therefore, four kinds of feature-fern combinations are tested, but the total feature number of RCS (300-D) and HOG-LBF(1000-D) is a constant. As is illustrated in Fig 4, the experiment shows that 30 ferns with size 10 for RCS feature and 100 ferns with size 10 for HOG-LBF feature yield the best results.

**Fig 5:**
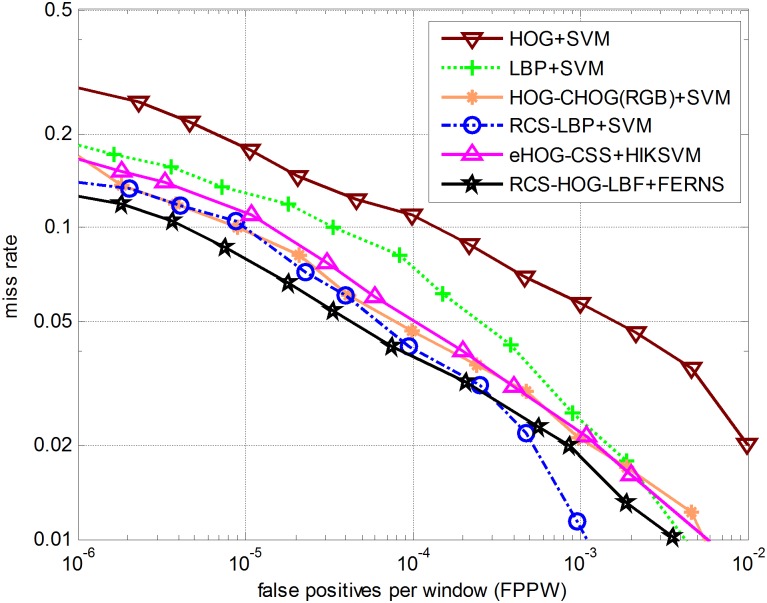
Comparison between the proposed method and five other methods.

Experiment results on the INRIA Person Dataset are presented using detection error tradeoff (DET) curves on a log-log scale. As displayed in Fig 5, the x-axis corresponds to false positives per window (FPPW), defined by *FalsePos/(TrueNeg + FalsePos)* and the y-axis shows the miss rate, defined by *FalseNeg/(FalseNeg + TruePos)*. Obviously low miss rate together with low FPPW is favorable.

We compare our proposed pedestrian detector with five other detectors such as Local Binary Pattern (LBP) feature combined with SVM classifier, Dalal & Triggs [3] proposed HOG feature and SVM classifier based detector, Zhang et al. [1] proposed Relative Color Similarity (RCS) feature fused with LBP feature, Ott and Everingham [19] explored HOG-CHOG feature and SVM classifier based detector, Zhao et al. [23] proposed Enhanced Histogram of Oriented Gradients (EHOG) fused with Color self-similarity (CSS) feature and Histogram Intersection Kernel SVM (HIKSVM) [30] classifier based detector. In addition, to rule out various unrelated factors and to highlight the distinctiveness of the feature itself, the experiments for comparing different features should avoid as many ad hoc optimizing tricks as possible, such as the detector being refined in an iterative process by selecting the most difficult examples (hard examples) from images. Detection error tradeoff (DET) for all descriptors is plotted in Fig 5.

As shown in Fig 5, we achieved the desired performance on the INRIA Person Dataset. For the six cases illustrated in Fig 5, the miss rates at 10^−4^ FPPW are approximately 11%, 6.9%, 5.0%, 4.6%, 4.0% and 3.8% from higher to lower, the miss rate based on our proposed detector reduces about 7.2% to initial HOG, and at least 0.2% to other five detectors. In comparison with other combinations of feature and classifier mentioned above, experiment results show that the detector with an augmented feature RCS-HOG-LBF and random ferns classifier achieves the best performance.

**Fig 6:**
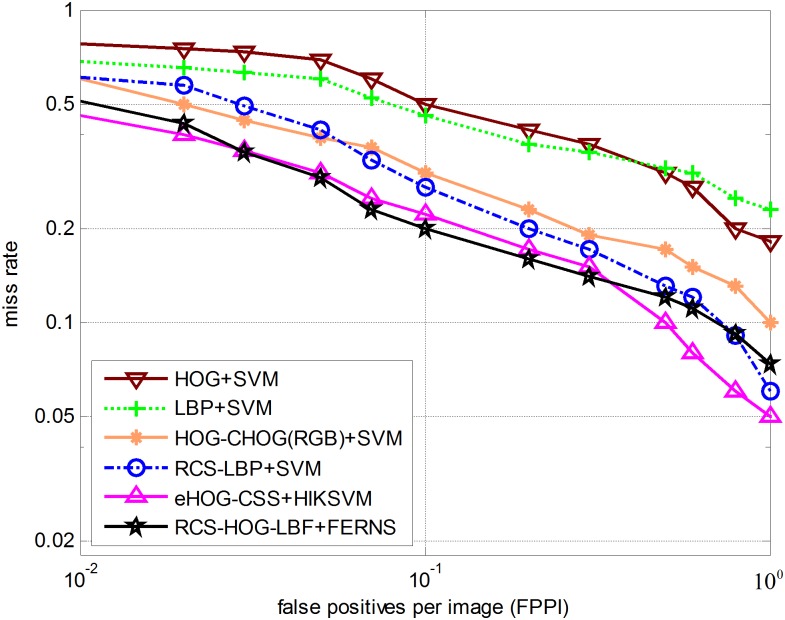
Performance comparison for different methods on the INRIA dataset.

The False Positives per Image (FPPI) miss rate evaluation method, which is also widely used in object detection, evaluates performance based on the detected images. As illustrated in Fig 6, we perform evaluation with respect to full images instead of detection windows introduced above; the detection rate improves by at least 2% (at 10^−1^ FPPI) compared to other five detectors. These experimental results demonstrate that our proposed method can effectively improve the detection rate.

**Fig 7:**
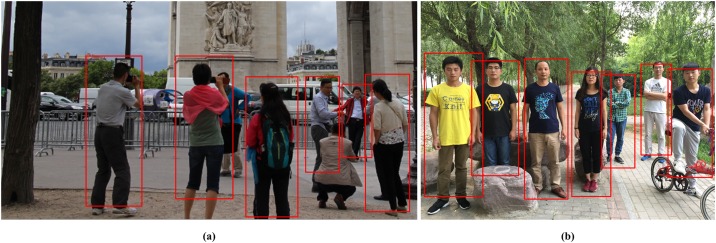
Sample detections on images densely scanned by the RCS-HOG-LBF detectors.

As illustrated in Fig 7, several sample detections on images were densely scanned by the RCS-HOG-LBF detectors. Fig 7(a) and 7(b) shows the crowded scene and various pedestrian postures. As we can see from Fig 7, the simulation results still show the superiority of our proposed approach, and offer a promising and significant human detection result. But, there are still some missing detections in Fig 7(a), for which, this is mostly due to the large posture change and highly occluded person that is at least 50% “invisible.

## Conclusions

In contrast with other detection targets, humans in standing positions have distinguishing characteristics, such as bilateral symmetry, strong vertical edges presented along the boundaries of the body, specific clothing textures, etc. Therefore, contour feature, texture feature and color feature can be extracted to detect pedestrians.

In this work, we presented an approach for human detection utilizing color-gradient based binary features as human descriptors and random ferns as classifier. The experimental results demonstrate that the superior performances in human detection are obtained by our proposed approach to the INRIA human database. In future work, we plan to explore further ways of utilizing color information, and new robust ways of encoding color as an additional feature, and attempt to refine the detector in an iterative process by selecting difficult examples such as pedestrian samples with large posture changes or heavy occlusion.
